# Cuproptosis patterns and tumor microenvironment in endometrial cancer

**DOI:** 10.3389/fgene.2022.1001374

**Published:** 2022-09-26

**Authors:** Junfeng Chen, Guocheng Wang, Xiaomei Luo, Jing Zhang, Yongli Zhang

**Affiliations:** ^1^ Department of Obstetrics and Gynecology, Shanghai First Maternity and Infant Hospital, Tongji University School of Medicine, Shanghai, China; ^2^ Department of Gynecological Oncology, The First Affiliated Hospital of Bengbu Medical College, Bengbu, China

**Keywords:** endometrial cancer, cuproptosis, tumor micoenvironment, immunothearpy, prognosis

## Abstract

Cuproptosis is the most recently discovered mode of cell death. It could affect the metabolism of cancer cells and surrounding infiltrating immune cells. In recent years, many studies have also shown that the tumor microenvironment (TME) plays a critical role in tumor growth and development. Mounting evidence suggests that Cuproptosis would bring unique insights into the development of pharmacological and nonpharmacological therapeutic techniques for cancer prevention and therapy. However, no study has been done on the combination of cuproptosis and TME in any cancer. Herein, we investigated the relationship between cuproptosis-related genes (CRGs), TME, and the prognosis of patients with Uterine Corpus Endometrial Carcinoma (UCEC). We identified three CRGs clusters based on 10 CRGs and three CRGs gene clusters based on 600 differentially expressed genes (DEGs) with significant prognostic differences. Following that, the CRGs score based on DEGs with significant prognostic differences was established to evaluate the prognosis and immunotherapeutic efficacy of UCEC patients. The CRGs score was shown to be useful in predicting clinical outcomes. Patients with a low CRGs score seemed to have a better prognosis, a better immunotherapeutic response, and a higher tumor mutation burden (TMB). In conclusion, our study explored the influence of cuproptosis patterns and TME on the prognosis of cancer patients, thereby improving their prognosis.

## Introduction

From plants to animals, all biology nearly needs metal ions to induce regulated cell death through different subroutines for normal health (Zhang et al., 2019). More or fewer of these metals can also play a role as the central gate-keeper for normal human functions (Zahn et al., 2021). In 2012, the concept of ferroptosis was discovered and proposed for the first time (Tang et al., 2021). Numerous investigations have been conducted over the last decade to investigate the mechanisms and roles of ferroptosis in cancer. (Proneth and Conrad, 2019; Jiang et al., 2021; Tang et al., 2021). It has great potential for tumor growth, drug resistance, and immune surveillance (Chen et al., 2021). In March 2022, Tsvetkov et al. (Tsvetkov et al., 2022) discovered a new mechanism of cell death that is copper-dependent, regulates cell death, differs from known death mechanisms, and relies on mitochondrial respiration. Additionally, they discovered that three genes (GLS, MTF1, and CDKN2A) sensitized the cells to cuproptosis whereas seven genes (LIAS, PDHA1, LIPT1, FDX1, DLD, DLAT, and PDHB) provided resistance to cuproptosis (Tsvetkov et al., 2022). Adenosine triphosphate (ATP) production is not necessary for cuproptosis; rather, it relies on mitochondrial respiration (Tsvetkov et al., 2022). Cuproptosis is caused by direct copper binding to lipoylated components of the tricarboxylic acid (TCA) cycle, lipoylated protein aggregation, and *Fe-S* cluster protein loss in mitochondria, all of which result in proteotoxic stress and eventually cellular death (Tang et al., 2022). Apoptosis, necroptosis, pyroptosis, ferroptosis, and other types of cell death, among others, were all connected to tumor development, metastasis, and immune treatment, according to earlier research (Andersen et al., 2005; Dannappel et al., 2014; Karki and Kanneganti, 2019; Lei et al., 2022).

In China, endometrial cancer is the most common gynecologic cancer, and its prevalence is increasing (Brooks et al., 2019). Despite the growing number of tumor treatments, the prognosis of advanced endometrial cancer has still not significantly improved (Ventriglia et al., 2017). Among the numerous cancer treatments, immunotherapy has emerged as a powerful clinical strategy for the treatment of cancer (Riley et al., 2019). Immune checkpoints have emerged as a new treatment strategy in oncology (O’Donnell et al., 2019). Many patients have resistance to immunotherapy, resulting in only a small percentage of patients benefiting from it (O’Donnell et al., 2019). Numerous studies have shown that the efficiency of immunotherapy is closely related to TME heterogeneity and metabolic plasticity (Li et al., 2019; Martin et al., 2020; Goliwas et al., 2021). The TME is comprised of multiple components, including chemokines, growth factors, exosomes, cytokines, and other molecules. So, copper can be a participant in the regulation of physiological and pathological processes in cancer (Li et al., 2014; Voli et al., 2020; Liu et al., 2021). Therefore, the investigation of inhibitors and regulators of copper-related variables as potential cancer therapeutics has gained increasing interest in the scientific community (Li et al., 2014; Garber, 2015; Voli et al., 2020; Liu et al., 2021).

However, there is a research gap on cuproptosis in UCEC. Therefore, there is an urgent need to comprehensively explore the prognostic significance of the cuproptosis and its association with TME infiltrating features in UCEC. Based on the advances in RNA sequencing, we mainly analyzed related-data from The Cancer Genome Atlas (TCGA) and Gene Expression Omnibus (GEO) by integrating multi-omics approaches.

## Materials and methods

### Data collection and processing

We downloaded the expression data, clinical information, and immunophenoscores (IPSs) for UCEC directly from TCGA (https://cancergenome.nih.gov/), GEO (https://www.ncbi.nlm.nih.gov/geo/), and the Cancer Immunome Atlas (TCIA) (https://tcia.at/home). The TCGA database contained information on 552 UCEC patients and 23 tumor-free individuals, and GSE17025, containing 80 UCEC samples, was downloaded from GEO. The TCGA also provided data on copy number variation (CNV) and somatic mutation data. To normalize gene expression data, the limma R package was used to convert fragments per kilobase per million (FPKM) values to transcripts per kilobase per million (TPM) values. R (*R version 4.1.1*) biological conductor packages were used to extract and analyze expression data and clinical information. Previous research (Tsvetkov et al., 2022) by Todd R. Golub’s team has identified 10 genes (FDX1, LIAS, LIPT1, DLD, DLAT, PDHA1, PDHB, MTF1, GLS, and CDKN2A) that are closely related to the occurrence and development of cuproptosis. Therefore, we selected these ten genes as CRGs for further research.

### Analysis of cuproptosis-related genes clusters based on 10 cuproptosis-related genes

The number of clusters and their stability were determined by consensus clustering. Based on the expression of 10 CRGs, we used unsupervised cluster analysis to classify all samples into three cuproptosis patterns with the optimal k value. We analyzed all samples using the “ConsensClusterPlus” R software package and ran cycle computation 1,000 to ensure classification stability. Principal component analysis (PCA) is used to estimate the distribution of molecular patterns. R package “GSVA” is a non-parametric and unsupervised method for estimating the gene set variation and activity change of biological processes in expression dataset samples (Hänzelmann et al., 2013). We used it to investigate the biological processes between different CRGs. We downloaded the gene set of “c2. cp.kegg.v7.5.1. symbols” from the MSigDB database (https://www.gsea-msigdb.org/gsea/msigdb/) for GSVA analysis. It was considered statistically significant when the adjusted *p*-value was < 0.05. The functions of CRGs were annotated using the “clusterProfiler” R program, with a critical value of false discovery rate (FDR) was < 0.05.

### Analysis of tumor microenvironment cell infiltration

We used single-sample gene-set enrichment analysis (ssGSEA) to quantify the relative abundance of each immune cell infiltration in the TME of the UCEC samples to better understand the degree of immune cell infiltration in the three CRGs clusters (Charoentong et al., 2017). The relevant gene set which distinguishes between infiltrating immune cell subtypes in TME was previously identified (Barbie et al., 2009; Charoentong et al., 2017). The relative abundance of different immune infiltrating cells, including activated DCs, activated CD8 T cells, macrophages, natural killer T cells, and regulatory T cells, in the TME in each sample was evaluated using the enrichment scores calculated by ssGSEA analysis.

### Analysis of cuproptosis-related genes clusters based on 600 differentially expressed genes with independent prognostic value

DEGs in nontumor tissues and UCEC samples were analyzed by the limma and ggplot2 packages, and the significance cutoff of DEGs was |logFC| > 1, *p* < 0.05 (Ritchie et al., 2015). The R package “VennDiatram” is used to construct Venn diagrams to identify common DEGs. Further, univariate Cox regression analysis identified the common DEGs with significant prognostic differences. Based on the expression of those DEGs with significant prognostic differences, the consensus clustering analysis was performed to determine the number of CRGs gene clusters resulting from biological variations. The Kaplan-Meier analysis was employed to perform the survival analysis between CRGs gene clusters.

### Construction of the cuproptosis-related genes score

To quantify the cuproptosis patterns of individual UCEC patients, we constructed a CRGs score system for UCEC patients. We selected 600 DEGs with significant prognostic differences to construct the CRGs score, and the principal components 1 and 2 were chosen as signature scores of cuproptosis patterns. The CRGs score was calculated as follows:

CRGs score=∑(PC1i+PC2i)

where *i* represents the expression of independent prognostic DEGs. Then, we separated all patients into high-and low-risk groups according to the CRGs score using the R package “ggalluvial.”

### Correlation between cuproptosis-related genes scores and immunotherapy

The IPS was extracted based on four categories of immunogenicity-related genes (MHC molecules, effector cells, immune modulators, and immunosuppressive cells). The expression of genes in different cell types is represented by the IPS score, which ranges from 0 to 10. Meanwhile, the values of IPS were positively correlated with immunotherapeutic efficacy by R package “ggpubr.”

### Statistical analysis

The correlation coefficients between the expression of 10 CRGs and infiltrating immune cells in TME were calculated by Spearman’s correlation analysis. The *t-*test and the Kruskal-Wallis test were used to compare two groups; one-way ANOVA and the Kruskal-Wallis test were used to compare three or more groups; and the Chi-square test and Fisher’s exact test were used to compare categorical variables. The Kaplan-Meier analysis with a log-rank test was used to plot the survival curve for the prognosis analysis. The waterfall diagram shows the mutation landscape using the maftools package. The *p-value* < 0.05 was considered statistically significant.

## Results

### The landscape (copy number variations, gene expression and mutation) of 10 cuproptosis-related genes in endometrial cancer

**FIGURE 1:**
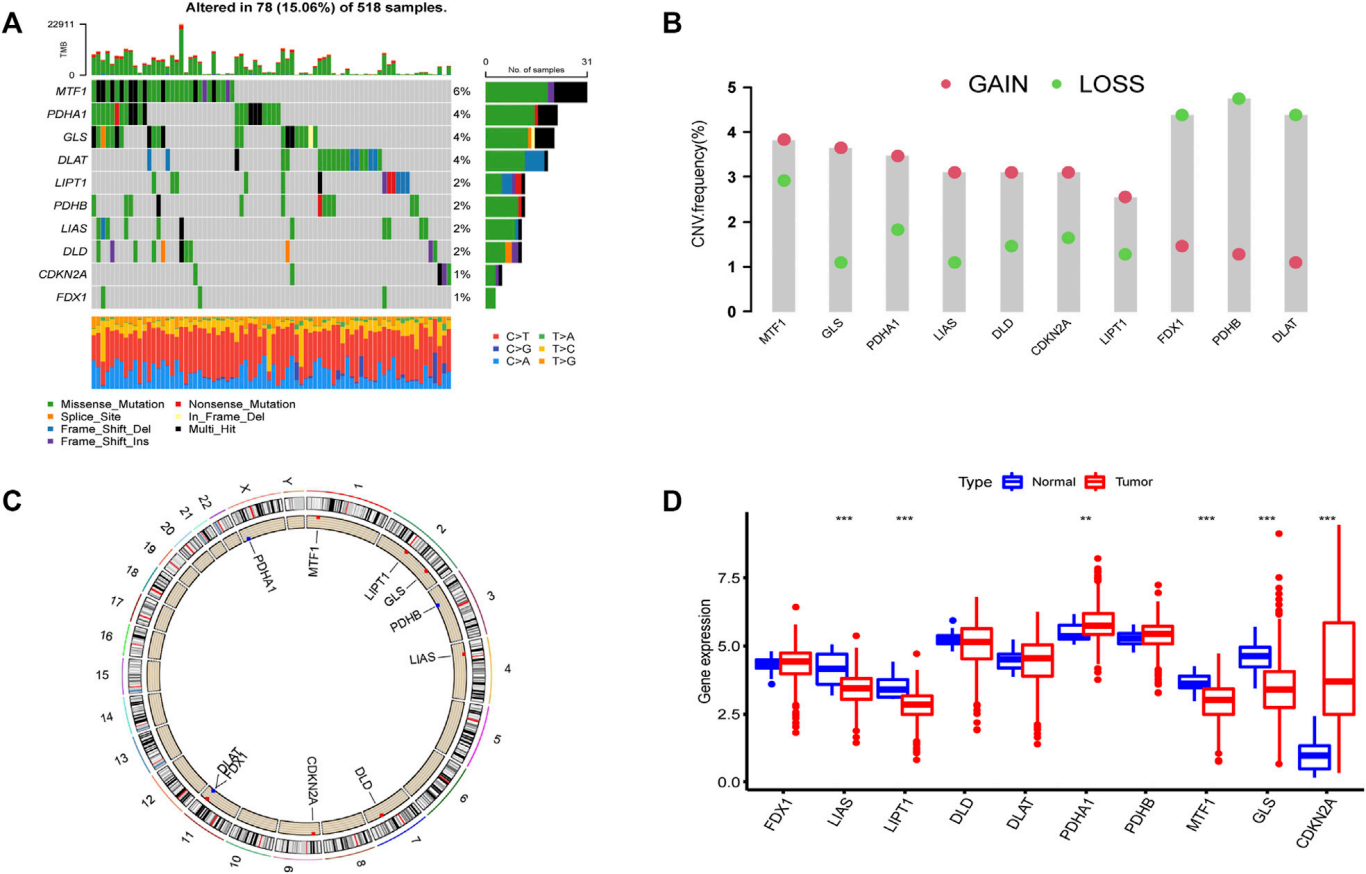
Landscape of 10 CRGs in endometrial cancer. **(A)** The mutation incidence of 10 CRGs in the TCGA-UCEC cohort. **(B)** CNV frequency of 10 CRGs in the TCGA-UCEC cohort. The gene deletion is shown by the green circle, the gene amplification is represented by the red circle, and the mutation frequency is shown by the height of the column. **(C)** The locations of the CNV of 10 CRGs on 23 human chromosomes. **(D)** The gene expression of 10 CRGs in normal (blue) and tumor tissues (red). The statistical *p*-value is shown by the above asterisk. (*p < 0.05, **p < 0.01, ***p < 0.001).

**FIGURE 2:**
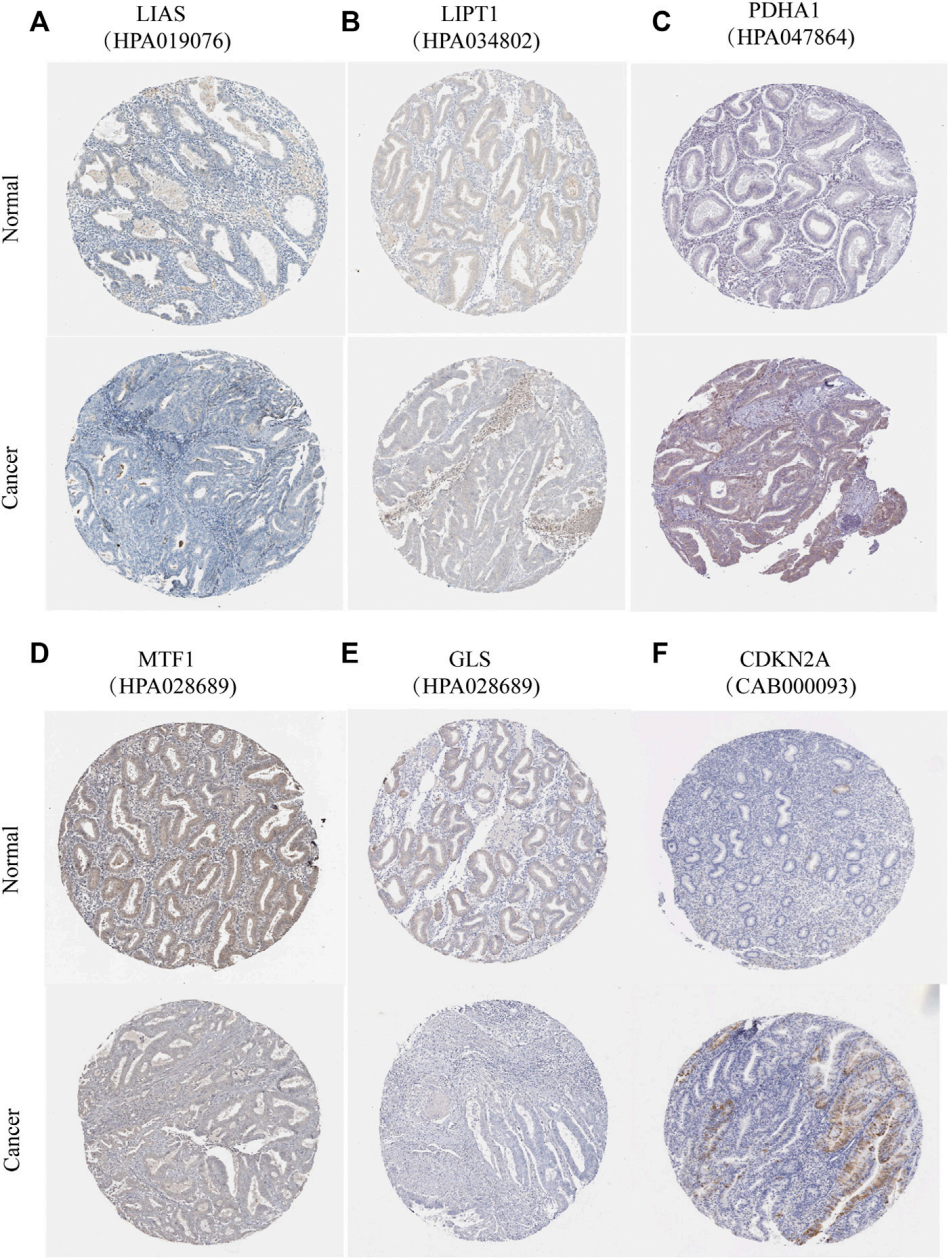
Protein expression of six CRGs in the HPA database. Verification of **(A)** LIAS **(B)** LIPT1 **(C)** PDHA1 **(D)** MTF1 **(E)** GLS **(F)** CDKN2A protein expression in normal and tumor tissue utilizing the Human Protein Atlas (HPA) database.

According to previous reports (Ge et al., 2022; Tsvetkov et al., 2022), a total of 10 CRGs were included for analysis in this study. Figure 1 summarizes the mutation rates of CNV and somatic mutations of CRGs. Only 78 (15.06%) of 518 samples caused mutations of CRGs (Figure 1A), but 10 CRGs were all mutated. Furthermore, the CNV frequency of 10 CRGs was shown to be common in UCEC, the majority of which were associated with copy number amplification (Figure 1B). However, *FDX1, PDHB,* and *DLAT* had a higher incidence of copy number deletion (Figure 1B). The locations of CNV alterations of 10 CRGs on the human chromosomes in the UCEC cohort are presented in Figure 1C. To investigate whether the aberrant expression were associated with UCEC, we analyzed the mRNA expression of CRGs in UCEC and normal tissues (Figure 1D). Most mRNA levels of CRGs differed considerably between normal and tumor samples (Figure 1D). Meanwhile, the protein expression (Figure 2) of the six CRGs in the normal and tumor groups was analyzed in the Human Protein Atlas (HPA) database (https://www.proteinatlas.org/). Supplementary Figure S1 revealed the expression of five genes (*LIPT1, DLD, PDHA1, GLS,* and *CDKN2A*) was strongly connected with the outcome of UCEC patients, and it demonstrated abnormal expression of CRGs was related to cancer carcinogenesis, development, and progression.

### Three different cuproptosis patterns are mediated by 10 cuproptosis-related genes

**FIGURE 3:**
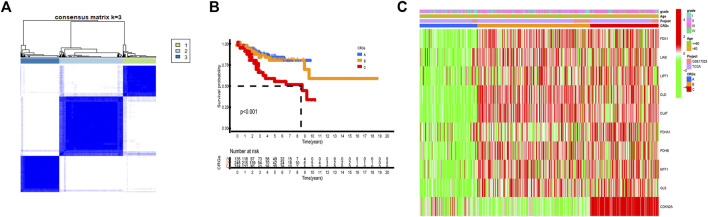
Three cuproptosis patterns and their clinical features. **(A)** Consensus clustering matrix of 10 CRGs for *k* = 3 using unsupervised clustering analysis. **(B)** Kaplan–Meier survival analysis of three cuproptosis patterns in the UCEC. *p*-value < 0.05 is considered a statistical difference among three cuproptosis patterns. **(C)** The heatmap presented the expression of 10 CRGs among three different cuproptosis patterns by unsupervised clustering in TCGA-UCEC and GSE17025 UCEC patients. The relationship among the CRGs cluster, grade, and age of 10 CRGs were used as notes. Red represented high expression and green represented low expression.

By carrying out unsupervised clustering analysis (Supplementary FigureS2), we classified all patients into different cuproptosis patterns according to the expression of the 10 CRGs. Finally, we discovered three different cuproptosis patterns named as CRGs clusters A, B, and C, including 154 cases in CRGs cluster A, 298 cases in CRGs cluster B, and 182 cases in CRGs cluster C (Figure 3A, Supplementary Table S1). The survival analysis of three cuproptosis patterns showed that CRGs cluster C had the worst survival situation (Figure 3B). After combing the TCGA-UCEC and GEO datasets, the relationship between clinical characteristics and gene expression of three different cuproptosis patterns was analyzed and exhibited in Figure 3C.

**FIGURE 4:**
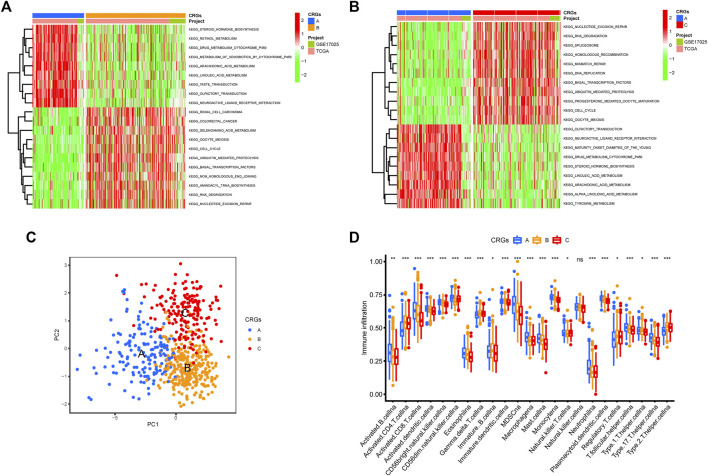
Biological pathways based on three clusters and the infiltrating immune cell component in TME. **(A,B)** The activation status of biological pathways in three CRGs clusters as depicted by GSVA enrichment analysis. The heatmap displayed in red represents activated pathways, and green represents inhibited pathways. **(A)** KEGG pathways of CRGs expression clusters A vs. B **(B)** KEGG pathways of CRGs expression clusters A vs. C. **(C)** Principal component analysis (PCA) shows significant differences among the three different cuproptosis patterns. **(D)** The expression abundance of infiltrating immune cells in three cuproptosis patterns. The boxplot showed the expression differences of 23 kinds of immune cells among CRGs cluster A, B, and C. The upper and lower ends of each box mean the quartile range of the value, the middle line represents the median value. The statistical *p*-value is shown by an asterisk (*p < 0.05, **p < 0.01, ***p < 0.001).

The GSVA enrichment analysis was used to investigate the enrichment of biological processes connected with the three types of cuproptosis patterns (Figures 4A,B). We found CRGs cluster A were significantly enriched in the pathways related to metabolism. CRGs cluster C was correlated with cell activity, including cell cycle, DNA replication and RNA degradation (Figures 4A,B). The transcriptome profiles of three cuproptosis patterns were analyzed using principal component analysis (PCA), which revealed substantial differences among the three clusters (Figure 4C). Subsequently, we evaluated the infiltrating immune cells in three different clusters. The ssGSEA analysis presented that CRGs cluster C was hardly enriched in the infiltration of innate immune cells, such as activated CD8^+^ T cells, activated CD4^+^ T cells, macrophages, mast cells, monocytes, natural killer cells, plasmacytoid dendritic cells, and T helper cells, etc. (Figure 4D). According to the results of our research, this might be the reason why patients with CRGs cluster C have the worst survival situation.

### Functional annotation of cuproptosis patterns

**FIGURE 5:**
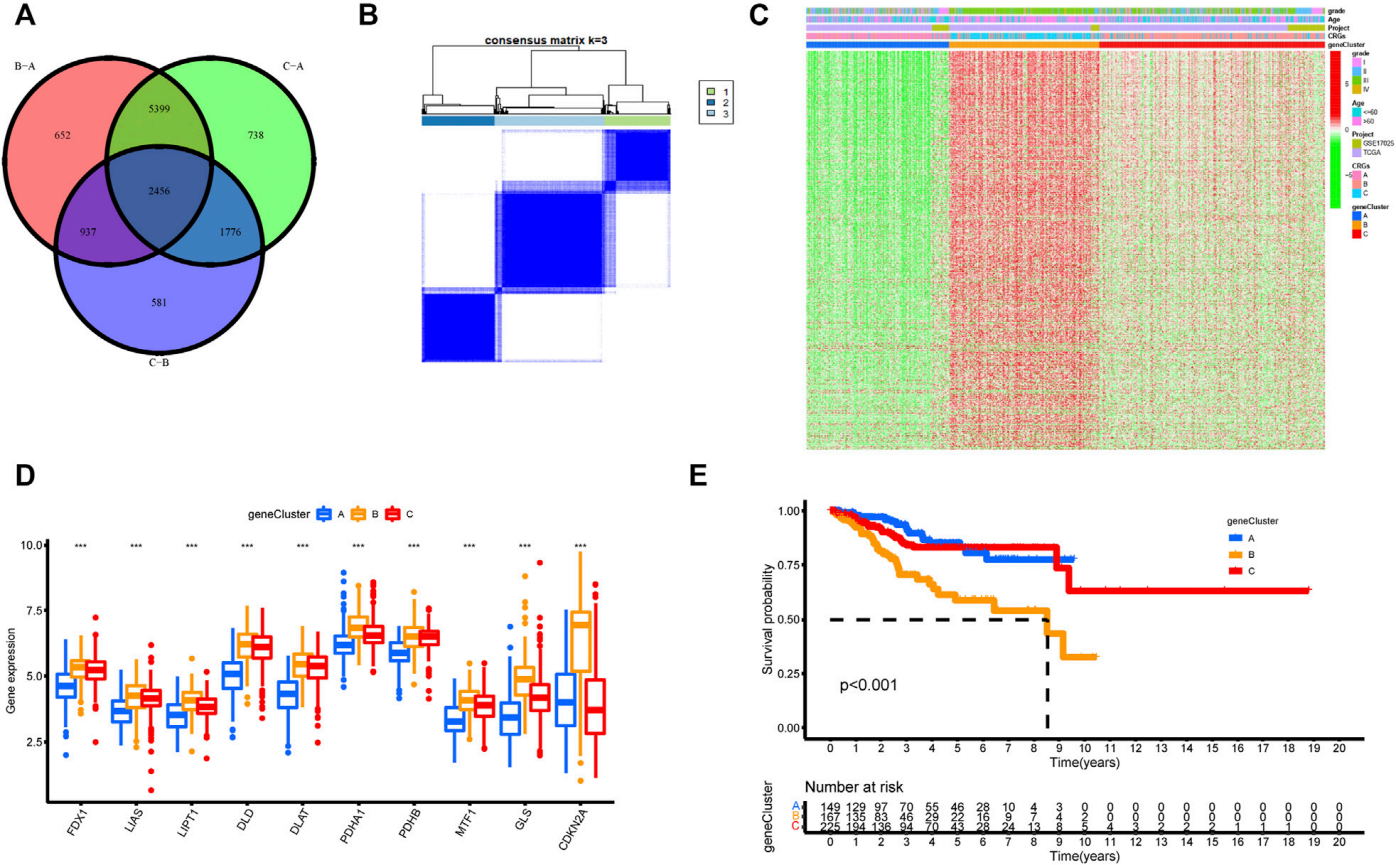
Unsupervised clustering of cuproptosis-related genes according to TCGA-UCEC and GSE17025 cohort. **(A)** 2,456 cuproptosis-related genes from TCGA are presented in the Venn diagram. **(B)** Consensus clustering matrix of cuproptosis-related genes for *k* = 3 using unsupervised clustering analysis. **(C)** The unsupervised clustering analysis of the CRGs gene clusters A, B, and C. CRGs clusters, CRGs gene clusters, grade, and age were noted. Red represented high expression, and green represented low expression. **(D)** The histogram illustrated the expression of 10 CRGs in three CRGs gene clusters. The upper and lower ends of each box mean the quartile range of the value, the middle line represents the median value. The statistical *p*-value is shown by an asterisk (*p < 0.05, **p < 0.01, ***p < 0.001). **(E)** Kaplan–Meier survival analysis for the three CRGs gene clusters (*p*-value < 0.05).

In order to further explore the potential biological regulatory pathways in the different cuproptosis patterns, we successfully identified 2,456 common DEGs from TCGA extracted from three distinct clusters associated with the cuproptosis patterns (Figure 5A). By univariate Cox regression analysis for OS, 600 DEGs with significant prognostic differences were selected for further study. The Gene Ontology (GO) and Kyoto Encyclopedia of Genes and Genomes (KEGG) function enrichment of these genes was analyzed using the clusterProfiler R package (Supplementary Figure S3). The GO analysis displayed that the DEGs were enriched in the regulation of cell cycle (Supplementary Figures S3A,B). The GO analysis of the biological process (BP) showed that these DEGs were enriched in autophagy, G1/S transition of mitotic cell cycle, and regulation of cell cycle phase transition. The cellular component (CC) exhibited that DEGs were abundant in the chromosomal region, mitochondrial matrix, and spindle. Molecular function (MF) indicated that DEGs mainly participated in catalytic activity, helicase activity, and ATPase activity. The KEGG analysis illustrated a significant enrichment of the cell cycle, protein processing in endoplasmic reticulum, autophagy, and ubiquitin mediated proteolysis (Supplementary Figures S3C,D).

### Identification and analysis of cuproptosis-related genes clusters

To further understand the regulatory mechanism of cuproptosis, we similarly used the limma package to divide all patients into distinct genotypes by unsupervised cluster analysis of the 600 cuproptosis-related DEGs filtered by univariate Cox regression analysis (Supplementary Table S2). We eventually identified three distinct CRGs gene clusters, including 169 cases in gene cluster A, 186 cases in gene cluster B, and 279 cases in gene cluster C (Figure 5B, Supplementary Table S3). Supplementary Figure S4 supported the three distinct CRGs gene clusters, named CRGs gene cluster A, cluster B, and cluster C, respectively. Figure 5C explored the clinical features of the three subtypes, and the three gene clusters were closely associated with CRGs clusters. In the three CRGs gene clusters, there were substantial differences in the expression of CRGs (Figure 5D). Once again, these findings affirmed that three diverse cuproptosis patterns occurred in UCEC. Furthermore, there was an observably bad prognosis in gene cluster B, which had a worse prognosis than CRGs gene clusters A and C (Figure 5E).

### Construction of the cuproptosis-related genes score and its correlation of tumor microenvironment and clinical prognosis

**FIGURE 6:**
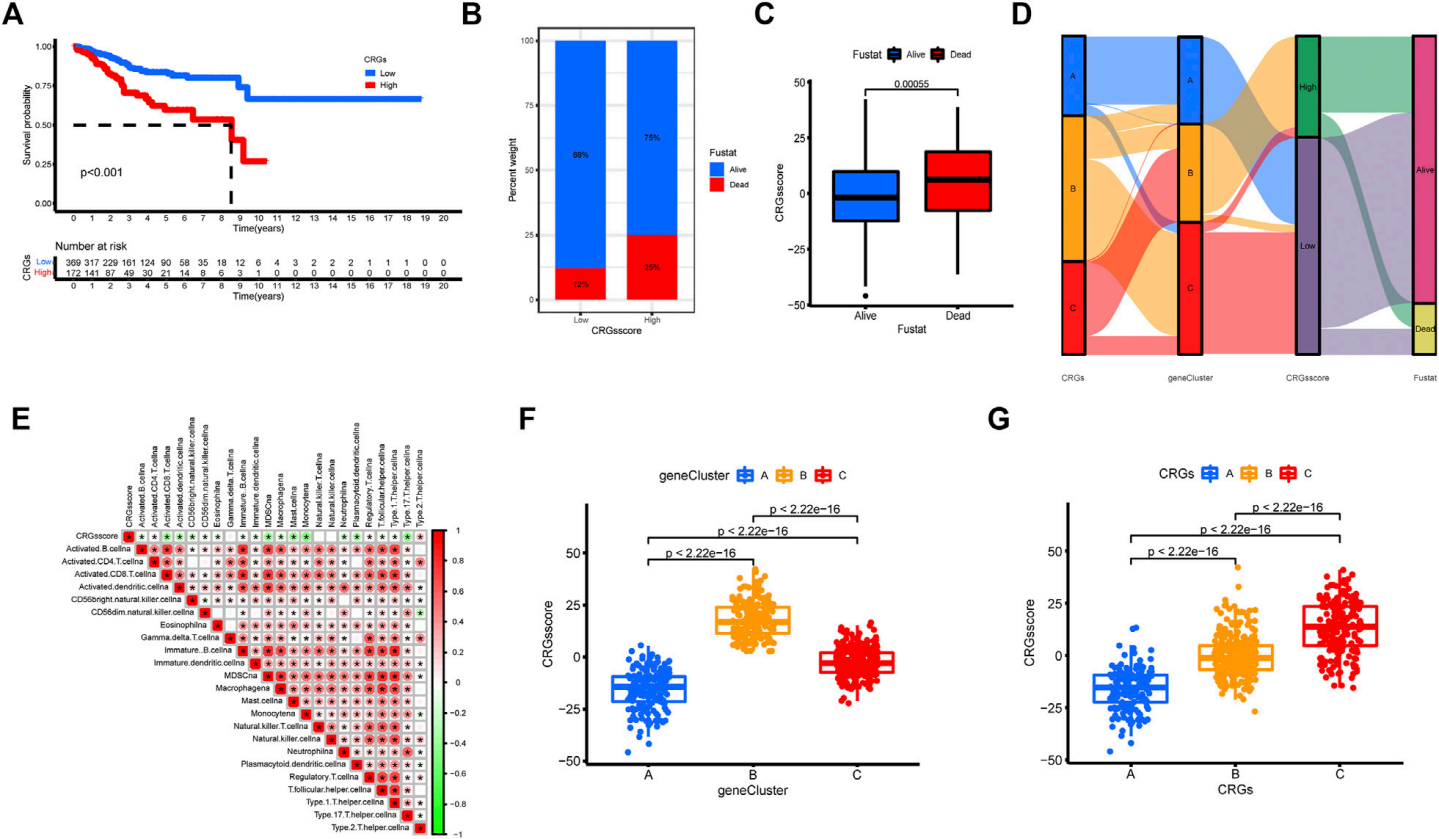
Characteristics of CRGs score. **(A)** Kaplan–Meier survival analyses for the OS in high and low CRGs score groups. **(B)** The percentage of patients in low and high CRGs score groups with various survival statuses. **(C)** The variation of CRGs score between the alive and dead group. **(D)** The alluvial diagram showed the relationship between CRGs cluster, gene cluster, CRGs score, and survival status. **(E)** Spearman analysis of the correlations between CRGs score and infiltrating immune cells. Red means the positive correlation and green means the negative correlation. The asterisk represents *p*-value that is meaningful statistically. **(F,G)** Differential analysis of CRGs score among **(F)** three CRGs gene clusters and **(G)** three CRGs clusters (*p* < 0.001).

In view of the various and complex alterations of CRGs in UCEC patients, we created a model that could quantify and evaluate cuproptosis patterns, namely the CRGs score. As shown in Figure 6A, patients with a low CRGs score had a higher survival rate than those with a high CRGs score. In the meantime, the high CRGs score group had a greater proportion of dead patients. According to the CRGs score, the CRGs score of alive patients was lower than that of dead patients (Figures 6B,C). To better show the features of the CRGs score, we evaluated the correlations between the CRGs score and immune infiltrating cells in TME. Only type 2 T helper cells were positively connected with the CRGs score, whereas other immune cells were negatively correlated (Figure 6E). According to the alluvial diagram (Figure 6D), the majority of the UCEC samples that indicated most patients with CRGs cluster C were grouped into CRGs gene cluster B, which obtained a high CRGs score and had lower survival rates. Furthermore, the Kruskal-Wallis test indicated significant differences in the CRGs score between the three CRGs gene clusters and the CRGs clusters (Figures 6F,G). In both of these studies, the higher CRGs score was found in CRGs cluster C and CRGs gene cluster B. (Figures 6E–G).

### Correlation between cuproptosis-related genes score and tumor mutation burden, prognosis, immunotherapy

**FIGURE 7:**
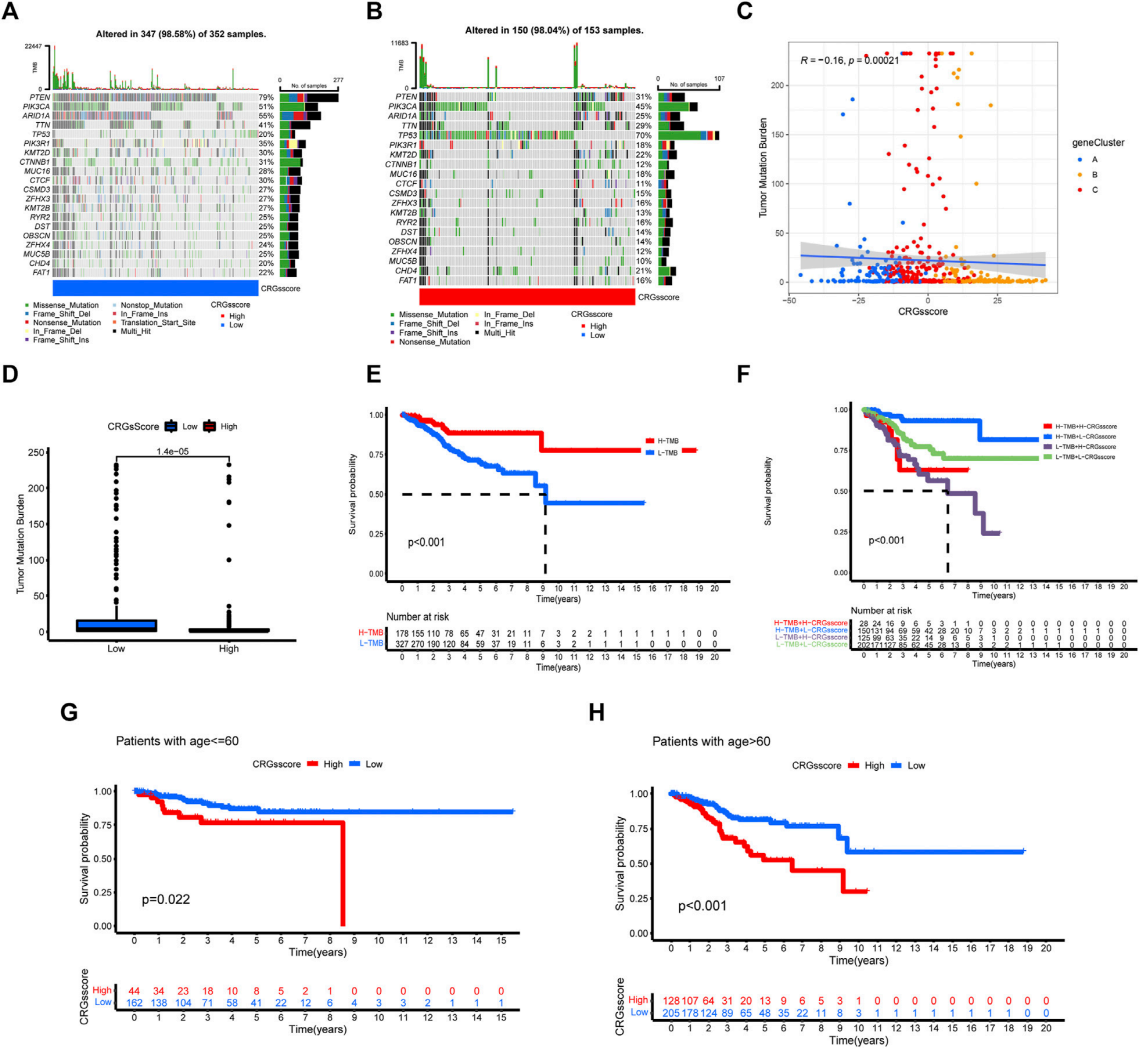
The relationship between CRGs score groups and somatic mutation, TMB. The waterfall plot of tumor somatic mutation in low CRGs score **(A)** and high CRGs score **(B)**. **(C,D)** The correlation between the CRGs score and TMB. **(E)** Survival analysis of CRGs score using Kaplan-Meier curves. **(F)** Survival analysis of both TMB and CRGs score using Kaplan-Meier curves. **(G,H)** Survival analysis of CRGs score in patients with age ≤ 60 and >60 using Kaplan-Meier curves.

We analyzed and visualized the differences in somatic mutation distribution profiles of UCEC patients in the high and low CRGs score groups by using the maftools package (Figures 7A,B). The low CRGs score group (98.58%) (Figure 7A) had a slightly higher proportion of somatic mutations than the high CRGs score group (98.04%) (Figure 7B). According to the quantitative study of TMB (Figure 7C), the CRGs score was found to have a negatively linear correlation with TMB. The TMB was higher in the low CRGs score group than in the high CRGs score group (Figure 7D), suggesting that low-risk patients may be more beneficial to immunotherapy. We next focused on the prognostic significance of TMB because of its importance. As seen in the survival plot (Figure 7E), those with high TMB had a greater prognosis benefit than patients with low TMB. Interestingly, merging the CRGs score and TMB may be used as a more comprehensive risk assessment (Figure 7F). In the meantime, we analyzed the prognosis between the CRGs score group and patients with age ≤ 60 and > 60 (Figures 7G,H). Regardless of patients with age ≤ 60 or > 60, the low CRGs score group has a better prognosis than the high CRGs score group (Figures 7G,H).

### Cuproptosis patterns in the role of immunotherapy

**FIGURE 8:**
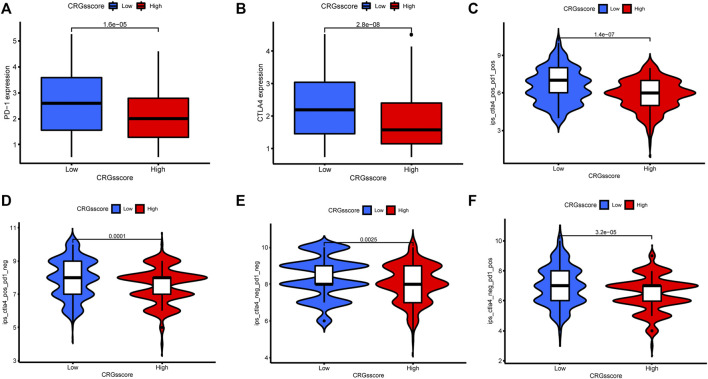
The association between CRGs score and immunotherapy. **(A,B)** The different expression of PD-1 and CTLA-4 in low CRGs score and high CRGs score. **(C–F)** Relationship between CRGs score and immunotherapeutic response of PD-1 and CTLA-4 expression: **(C)** positive PD-1 and CTLA-4. **(D)** Positive CTLA-4 and negative PD-1. **(E)** Negative PD-1 and CTLA-4. **(F)** Negative PD-1 and positive CTLA-4.

Figures 8A,B exhibited that patients with a low CRGs score had much greater PD-1 and CTLA-4 expression, indicating a possible great response to anti-PD-1 and anti-CTLA-4 immunotherapy. Simultaneously, we utilized the CRGs score to predict how people will react to the effectiveness of anti-CTLA-4 and anti-PD-1 immunotherapy. Patients with low CRGs scores (Figures 8C–F) had considerably better anti-CTLA4 and anti-PD1 treatment responses. In addition, IPS was obviously higher in the low CRGs score group than in the high CRGs score group for the combination of anti-PD1 and anti-CTLA-4 (Figure 8C). In general, these findings consistently show that the low CRGs score group has better immunotherapeutic effectiveness than the high CRGs score group. The findings also showed that the cuproptosis pattern is strongly associated with TMB and PD-1/CTLA-4 immunotherapy.

## Discussion

In recent years, a large number of studies (Singh et al., 2020; Cui et al., 2021; Guo et al., 2021; Ge et al., 2022) have found that copper plays a crucial role in the occurrence and development of human tumors. Copper can impact essential cellular processes by acting as both a negative allosteric regulator and a positive allosteric regulator of enzyme function (Ge et al., 2022). Cell proliferation and autophagy pathways are all affected, which eventually leads to tumor initiation and development (Ge et al., 2022). Therefore, inhibitors and regulators of cuproptosis-related genes have been investigated as potential methods of cancer therapeutics (Skrott et al., 2017; Yang et al., 2022). However, CRGs have been poorly studied in endometrial cancer and its association with immune infiltration in TME.

Simultaneously, according to the traditional histopathological subtypes, *PTEN* inactivation is a major driver of endometrioid carcinomas; *TP53* inactivation is a major driver of most serous carcinomas, some high-grade endometrioid carcinomas, and many uterine carcinosarcomas; and inactivation of either gene is a major driver of some clear cell carcinomas (Bell and Ellenson, 2019; Urick and Bell, 2019). Integrated genomic analysis by TCGA resulted in the molecular classification of endometrioid and serous carcinomas into four distinct subgroups: POLE (ultramutated), microsatellite instability (hypermutated), copy number low (endometrioid), and copy number high (serous-like) (Bell and Ellenson, 2019).

However, these existing classifications based on these molecules did not better improve the prognosis of UCEC. Molecular patterns based on molecular pathology can represent deeper properties of tumors and, as a result, compensate for the shortcomings of the traditional histopathological subtypes. The major purpose of our study is to identify three cuproptosis-related molecular patterns, construct the CRGs score based on 10 CRGs and investigate its correlation with the tumor immune microenvironment in UCEC. Moreover, our research found a connection between the CRGs score and immunotherapy, especially anti-PD-1/PDL1 and CTLA-4 immunotherapy.

First, we analyzed transcriptional sequencing data from GEO and TCGA-UCEC to identify the cuproptosis mode in UCEC. In our study, we successfully divided all samples into three cuproptosis-related molecular patterns based on 10 CRGs in UCEC. The common DEGs among the three molecular patterns were then discovered, and common DEGs filtered by univariate Cox regression analysis were then utilized to build the CRGs score. The KM survival curve illustrated a better OS and better prognosis associated with a low CRGs score than with a high CRGs score. The CRGs score might be used to identify different immunological phenotypes and assess the immunotherapy effects of UCEC patients.

The TME is associated with regulating cancer as well as a source of immune therapeutic targets. So, we explored the compositions of infiltrating cells in TME that were different in three patterns. Extensive articles (Tan et al., 2018; Hessmann et al., 2020; Jin and Jin, 2020; Bejarano et al., 2021; Singleton et al., 2021) reported that the TME plays a significant role in cancer development and might be a therapeutic target as well as a regulator of cancer development. We focused on the role of the CRGs of TME, with the goal of revealing its possible roles and contributing to a better understanding of the antitumor immunotherapy effects in UCEC. CD8^+^ T cells are the major anti-tumor effector cells (Mami-Chouaib et al., 2018), and T cell-mediated antitumor immunity is strengthened by tumor-infiltrating B cells (Mami-Chouaib et al., 2018; Engelhard et al., 2021). Natural killer (NK) cells can swiftly kill adjacent tumor cells, enhance antibody and T cell response as anticancer agents (Shimasaki et al., 2020). Expansion and genetic modification of NK cells can greatly increase their anti-tumor activity, and NK cells are expected to become essential elements of multipronged therapeutic strategies for cancer (Myers and Miller, 2021). Both innate and adaptive immune responses are mediated by dendritic cells (DCs) that play an important role in the progression and regulation of both innate and adaptive immune responses (Wculek et al., 2020). Due to the lower expression of almost all immune cells in CRGs cluster C, it might be the reason that the CRGs cluster C showed a lower survival rate than other clusters.

Besides, copy number variation (CNV) is one of the most important somatic aberrations in cancer, and it contributes to the pathogenesis of many disease phenotypes (Martin-Trujillo et al., 2017). Based on 10 CRGs and UCEC copy-number profiles, we explored the alteration of CRGs in UCEC. Mutations in the 10 CRGs were relatively uncommon, but CNV amplification and deletion were common events. Due to the specific function of PD-1/PD-L1 blockage immunotherapy in the field of cancer therapy, PD-1 expression of immune cells was examined independently for PD-1 immune treatment, and the IPS was developed as a marker to distinguish factors in clinical (Kalbasi and Ribas, 2020). While TMB analysis showed the low CRGs score group had a higher TMB than the high CRGs score group, several lines of evidence (Samstein et al., 2019; Marabelle et al., 2020; Valero et al., 2021) found the high TMB is associated with better survival for tumor patients receiving immune checkpoint inhibitors (ICIs). Our CRGs score system revealed that a combination of CTLA-4 and PD-1 blockers might be beneficial for the treatment of UCEC. A similar conclusion was found in some studies (Liu and Zamarin, 2018; Oh and Chae, 2019; Rotte, 2019; Zhang et al., 2021). The CRGs score can also be predicted to determine how tumors will respond to immunotherapy.

Based on the expression of CRGs, we discovered three molecular patterns connected to cuproptosis in the current study. In order to forecast patient survival and TME features, we created the CRGs score for each patient. In the meanwhile, this study demonstrated how CRGs affect the prognosis of UCEC patients. The CRGs score performs well in determining biological state and predicting UCEC survival. However, our research still has many shortcomings and flaws. First, many experiments have not yet been performed since cuproptosis was discovered not long ago. Therefore, there are no more basic experiments to demonstrate the effect of cuproptosis on the prognosis of endometrial cancer. Second, the mechanism by which cuproptosis affects cancer progression has not been revealed. Therefore, more research by well-designed experiments is needed.

## Conclusion

This study implies that CRGs are involved in the formation and progression of endometrial cancer. Based on the expression of 10 CRGs, we classified all samples into three cuproptosis patterns. According to 600 cuproptosis-related DEGs filtered by univariate Cox regression analysis, the CRGs score was constructed. The CRGs score was correlated with the tumor microenvironment, immunotherapy response, and prognosis of cancer patients. Through comprehensive analysis of the relationship between the CRGs score and clinicopathological features, we found it may help assess the efficacy of ICIs. In summary, the identification of cuproptosis patterns and related genes will contribute to valuable approaches for individualized therapy for UCEC patients.

## Data Availability

The original contributions presented in the study are included in the article/Supplementary Material, further inquiries can be directed to the corresponding authors.
