# Supplementary material for: Cuproptosis patterns and tumor microenvironment in endometrial cancer
Source: Front Genet. 2022 Sep 26;13:1001374. doi: 10.3389/fgene.2022.1001374 (PMC9549213; doi:10.3389/fgene.2022.1001374)
Supplement: Supplementary file 3 [file DataSheet1.doc]

**Cuproptosis** **Patterns and Tumor Microenvironment in Endometrial Cancer**

**Junfeng Chen**^1^**, Guocheng Wang**^1^**, Xiaomei Luo^1^, Jing Zhang**^2^***, Yongli Zhang^1^***

*^1^Department of Obstetrics and Gynecology, Shanghai First Maternity and Infant Hospital, Tongji University School of Medicine, Shanghai, China*

*^2^Department of Gynecological Oncology, The First Affiliated Hospital of Bengbu Medical College, Anhui, China*

Correspondence should be addressed to Yongli Zhang; [doctorzhang2@163.com](mailto:doctorzhang2@163.com) and Jing Zhang; [zhangjing1179@163.com](mailto:zhangjing1179@163.com)

Supplementary Table S1 | Classification of the three different CRGs clusters.

Supplementary Table S2 | 600 cuproptosis-related DEGs with independent prognostic analysis

Supplementary Table S3 | Classification of the three different CRGs gene clusters.


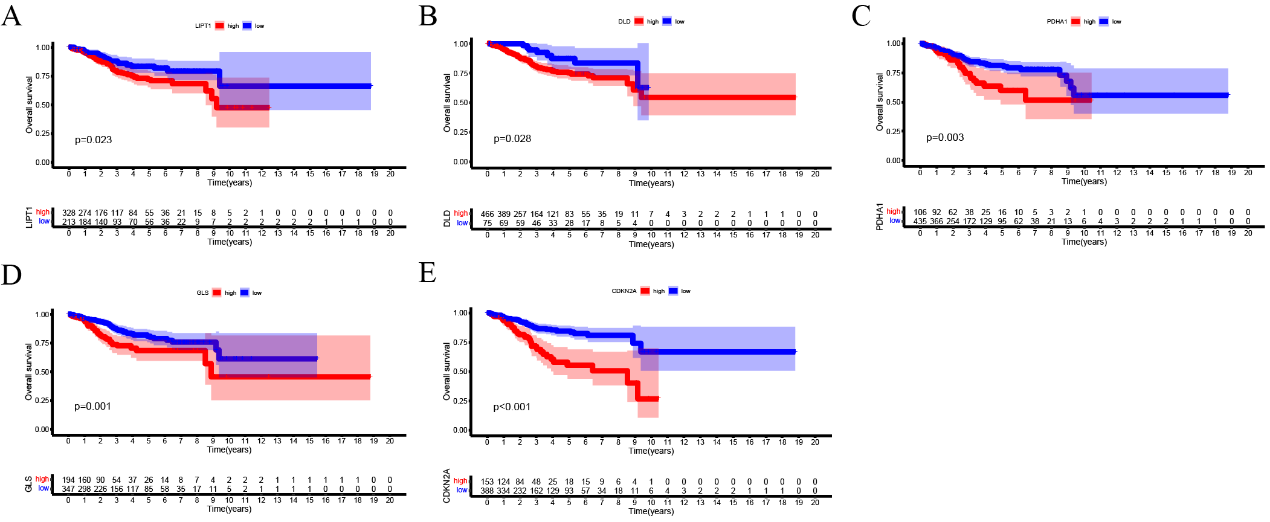


Supplementary Figure S1 | Survival analysis of 5 CRGs (*p* < 0.05).


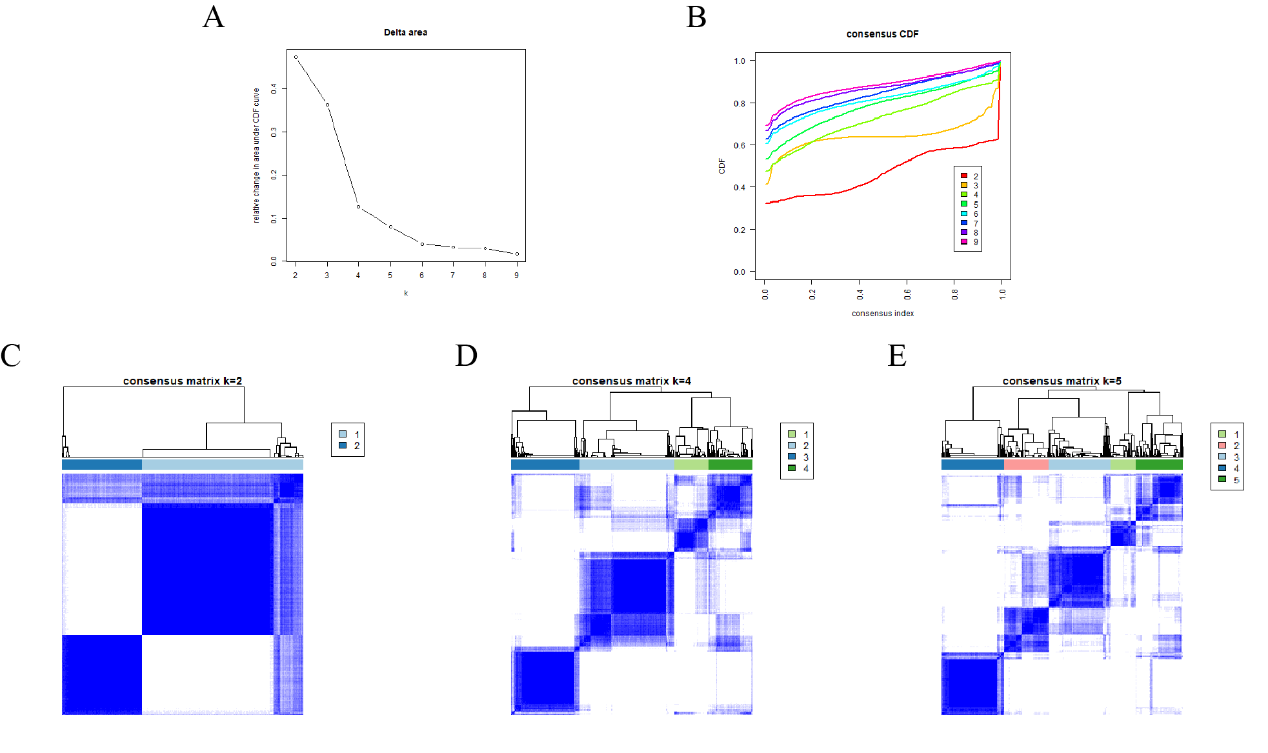


Supplementary Figure S2 | Consensus clustering matrices for k = 2-5 in different CRGs clusters.


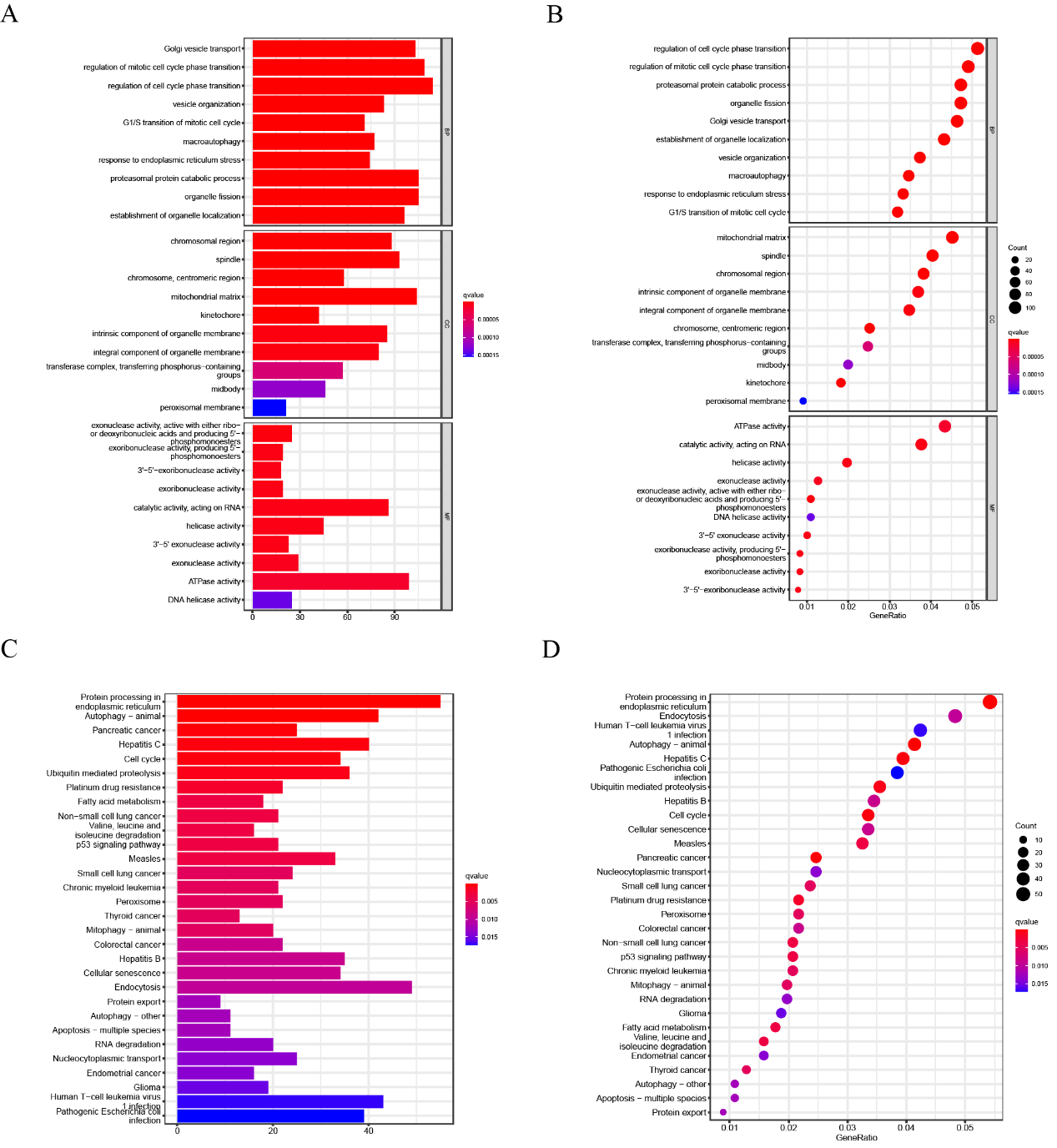


Supplementary Figure S3 | The GO functional annotation (A, B) and KEGG enrichment analysis (C, D) are analyzed for the cuproptosis related-genes.


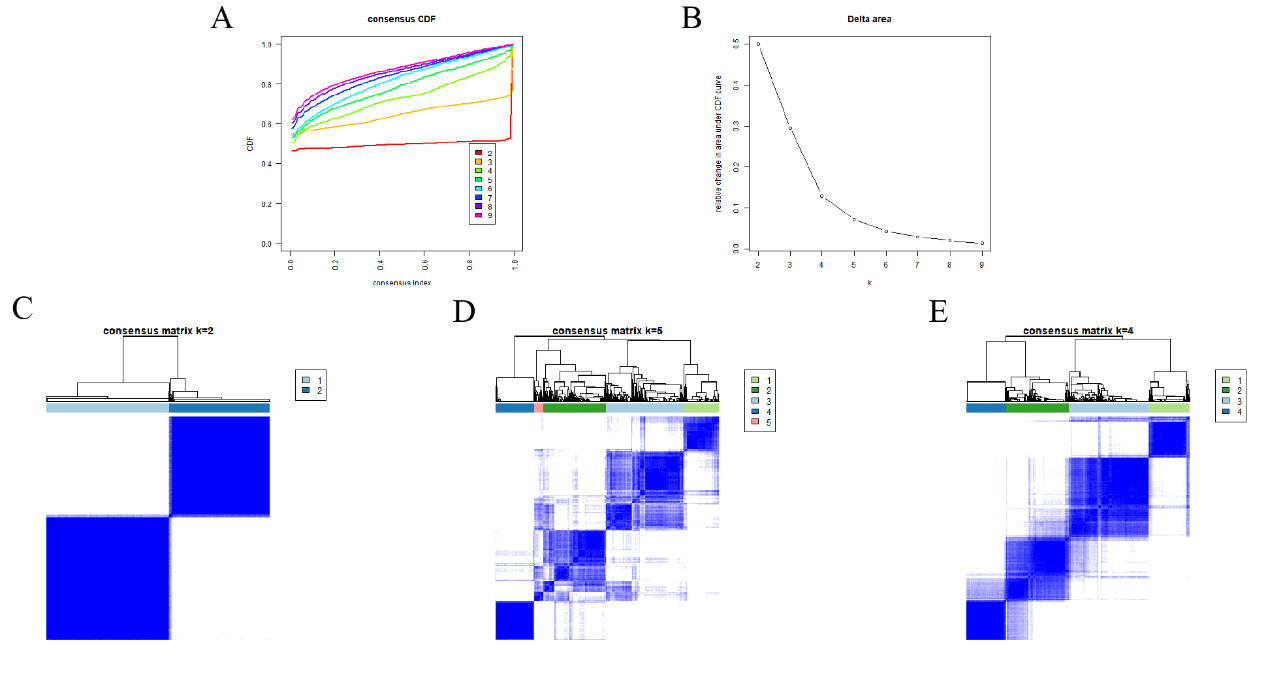


Supplementary Figure S4 | Consensus clustering matrices for k = 2-5 in different CRGs gene clusters.
