# Supplementary material for: Cuproptosis patterns and tumor microenvironment in endometrial cancer
Source: Front Genet. 2022 Sep 26;13:1001374. doi: 10.3389/fgene.2022.1001374 (PMC9549213; doi:10.3389/fgene.2022.1001374)
Supplement: Supplementary file 5 [file DataSheet2.docx]

https://www.jianguoyun.com/p/DQYK2PkQ4uflChjvj88E
